# How the initial level of visibility and limited resource affect the evolution of cooperation

**DOI:** 10.1038/srep27191

**Published:** 2016-06-02

**Authors:** Dun Han, Dandan Li, Mei Sun

**Affiliations:** 1 Nonlinear Scientific Research Center, Jiangsu University, Zhenjiang, Jiangsu, 212013, PR China; 2 College of Economics and Management, Nanjing University of Aeronautics and Astronautics, Nanjing, Jiangsu, 211106, China

## Abstract

This work sheds important light on how the initial level of visibility and limited resource might affect the evolution of the players’ strategies under different network structure. We perform the prisoner’s dilemma game in the lattice network and the scale-free network, the simulation results indicate that the average density of death in lattice network decreases with the increases of the initial proportion of visibility. However, the contrary phenomenon is observed in the scale-free network. Further results reflect that the individuals’ payoff in lattice network is significantly larger than the one in the scale-free network. In the lattice network, the visibility individuals could earn much more than the invisibility one. However, the difference is not apparent in the scale-free network. We also find that a high Successful-Defection-Payoff (SDB) and a rich natural environment have relatively larger deleterious cooperation effects. A high SDB is beneficial to raising the level of visibility in the heterogeneous network, however, that has adverse visibility consequences in homogeneous network. Our result reveals that players are more likely to cooperate voluntarily under homogeneous network structure.

Game theory on networks has attracted much interest from physicists in the last decade^1^,^2^,^3^, both because of the new phenomena that such a non-Hamiltonian dynamics gives rise to, and because of its very many important applications^4^,^5^,^6^,^7^. Game theory provides a powerful framework to understand the ubiquitous cooperative behaviors in social and biological systems^8^,^9^,^10^. People could understand the origin of the emergence and persistence of cooperation among selfish individuals by using game theories. Of more interest is what happens when individual payoff depends on the actions of others^11^. Although cooperation is ubiquitous in social life and an important topic for all kinds of economic interaction, field data rarely allow for a clean discrimination among competing theories. Because carefully designed laboratory experiments do allow for such rigorous comparisons, laboratory experiments have provided numerous important insights into cooperative behavior, and the resulting rich literature forms the basis of most of our knowledge on human cooperation^12^.

Under the complexity of human mobility and interaction, the individual’s decision whether or not to cooperate could evolve in time depending on their surroundings^13^,^14^,^15^. However, in many real-world social systems, individuals could not access to their surroundings, such as others’ payoff or wealth histories^16^. That is to say, people know their own information, but they could not get any information about their directly connected neighbors. In general, the individuals who are only aware of their own circumstances but do not know their neighbors, we named them “invisibility”. However, the individuals who are not only aware of their own situation but also know their neighbors, we call them “visibility”. To investigate some of the possible determinants and consequences of visibility, by using game method, the researchers who come from Yale University performed experiments involving a networked public goods game in which subjects interact and gain or lose wealth. Their results claimed that wealth visibility makes more people cooperation than when wealth is invisible and wealth visibility leads to greater inequality than when wealth is invisible^17^,^18^.

The famous prisoner’s dilemma game (PDG) model describes the conflict among cooperation and defection^19^,^20^,^21^,^22^,^23^. This model in particular has become a standard model for studying cooperation and cheating, with cooperation often emerging as a robust outcome in evolving populations^24^,^25^,^26^,^27^,^28^,^29^. Therefore, we just cannot help asking how the initial visibility affects the players in the prisoner’s dilemma game. In nature, the environment condition impels the resource distributing to each individual is limited. However, such condition creates opportunities for the smart players. Wise men could obtain more materials according to adopting some properly strategies. Based on the Darwin’s theory of evolution, therefore, the losers who own less resource are more likely to be replaced.

In this paper, we shed light on the question that how the initial level of visibility and limited resource affect the individuals’ strategies. Considering the limited resource, individuals distribute the total of resource according to their game payoff. The principle of “survival of the fittest” is introduced to update the players. At each update, therefore, the one whose resource amount is lower than critical survival value will be replaced by the offspring of its neighbor (those replaced players are called death). The Monte Carlo method is used to model the evolutionary game process. The simulation results indicate that the average density of death in lattice network decreases with the increases of the initial proportion of visibility. However, the contrary phenomenon is observed in the scale-free network. Further results present the individuals’ payoff in lattice network is significantly larger than the one in the scale-free network. The visibility individuals obtain more payoff than invisibility one in the lattice network. However, the difference is not apparent in the scale-free network. We also find that a high Successful-Defection-Payoff (SDB) and a rich natural environment have relatively larger deleterious cooperation effects. A high SDB is beneficial to raising the level of visibility in the heterogeneous network, however, that has adverse visibility consequences in homogeneous network. Our result reveals that players in homogeneous network are more likely to cooperate voluntarily than the one in heterogeneous network.

In what follows, we present the results, where we first introduce the evolutionary prisoner’s dilemma game with payoff visibility and then deliver our main conclusions. Lastly, we detailed discuss and conclude our results.

## Results

In the classical Prisoner’s dilemma game, each player could decide to be a cooperator or a defector simultaneously. If a cooperator links with a defector, the former player only obtains a payoff *S*, while the latter gets payoff *T* (we named it as the Successful-Defection-Payoff). Mutual cooperation makes both players get the payoff *R*, and mutual defection brings each player the payoff *P*. For the prisoner’s dilemma game thus satisfy the conditions: *T* > *R* > *P* > *S* and 2*R* > *T* + *S*. *R* > *P* means that mutual cooperation products higher payoff than mutual defection. The condition *T* > *R* and *P* > *S* mean that a player with defection strategy will obtain higher payoff, regardless of what choice the opponent makes in a single round of the game. Moreover, 2*R* > *T* + *S* is required to ensure that the cooperation would be not disappeared. In this paper, we denote the strategy of player *i* is *s*_*i*_. If the player *i* chooses to be a cooperator, then *s*_*i*_ = 1, otherwise, *s*_*i*_ = −1. The payoff matrix of the prisoner’s dilemma game can be depicted as shown in Table 1.

Assumption that initial proportion of visibility is *β*. Those visibility individuals could get know their own payoff as well as the payoff of each of their directly connected neighbors. However, the invisibility individuals could only acquire their own payoff. Considering the players’ visibility, a player might have the following two selections of strategy:

1. A visibility player *i* will decide whether or not to adopt his neighbors’ strategy. The player *i* with strategy *s*_*i*_ will choose the neighbor *j* who has maximum payoff and imitate the strategy of *j*. We apply the Fermi rule, namely an individual *i* will adopt the selected neighbor *j* strategy with probability:


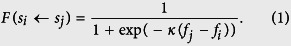


where *κ* denotes the amplitude of noise. For a small *κ*, individuals are less responsive to payoff difference, and the strategy of the individual with a high payoff becomes less likely to be adopted. Without losing generality, we set *κ* = 10, meaning that it is very likely that the better performing players will pass their strategy to their neighbors, yet it is also possible that players will occasionally learn from a less successful neighbor.

2. An invisibility player *i* will decide the next round game strategy mainly based on his own experiment. Specifically, if a player could benefit a lot using the adopted strategy at round *t*−1, then the player *i* will continue to retain the same strategy. However, if the adopted strategy at round *t*−1 could benefit a little, then the player *i* will adopt the opposite strategy. The adopted strategy of player *i* at *t* game round could be described as follows:


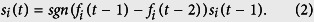


Here, we use −*s*_*i*_ implying the opposite strategy of *s*_*i*_. *f*_*i*_(*t* − 1) and *f*_*i*_(*t* − 2) are the individual’s payoff at game round *t* − 1 and *t* − 2, respectively. Typically, the player *i* will choose to be a cooperator or a defector randomly if *f*_*i*_(*t* − 1) = *f*_*i*_(*t* − 2).

The total resource of the system is *G* = *rN*, where *N* is the total number of players, *r* > 0 is the environment parameter. In general, a relative larger *r* means a rich and generous natural environment. After each game round, all players could obtain the resource based on their payoffs. The resource distributed into individual *i* is proportional to the player’s payoff. Therefore, the player *i* with payoff *f* could get the total resource *D*_*i*_ as follows:


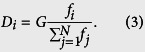


The Eq. (3) indicates that the smart players in the games could get higher payoff. After subtracting the consumption, the eventually resource for player *i* is *L*_*i*_ = *D*_*i*_ − 1.

According to the Darwin’s theory of evolution: “all species of organisms arise and develop through the natural selection of small, inherited variations that increase the individual’s ability to compete, survive, and reproduce”, therefore, the principle of “survival of the fittest” is considered. Specifically, the one whose resource amount *L*_*i*_ is lower than critical survival value *δ* will be replaced by offspring of its neighbor. The offspring have the same visibility and strategy with their ancestors. However, if all of its neighbors are eliminated, a randomly selected player’ offspring would replace the death one.

**Figure 1:**
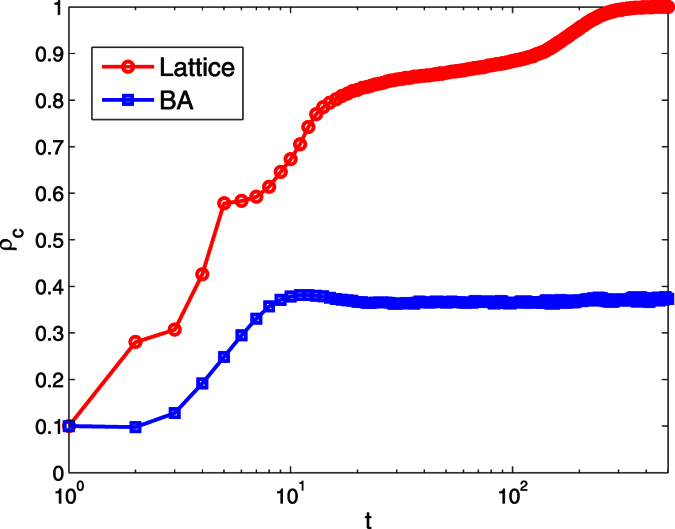
The evolutions of cooperator density in the lattice network and the BA network. As the game round goes, the density of cooperator increases in both two networks. However, the density of cooperator in scale-free network is less than in the lattice network. Parameters are set: *α* = 0.1, *β* = 0.5, *r* = 1.1, *δ* = 0, *T* = 1.1, *κ* = 10. Each data point is obtained by averaging 100 independent runs.

Figure 1 illustrates the evolutions of cooperator density *ρ*_*c*_ in the lattice network and the BA network. As the game round goes, the density of cooperator increases in both networks. However, the density of cooperator in scale-free network is less than in the lattice network. This result indicates that under limited resource and payoff visibility, homogenous network structure could promote the cooperation. Moreover, before the cooperator density reaches the stable-state, it needs last more game rounds in lattice network than in scale-free network. By calculating the diameter of the two networks (see the methods), we could find the BA network diameter *D* = 8 is far smaller than the lattice network diameter *D* = 98. Therefore, the “thought” (whether or not to adopt the cooperation) diffuses fast in the scale-free network, which leads to a less game rounds to reach the stable-state.

**Figure 2:**
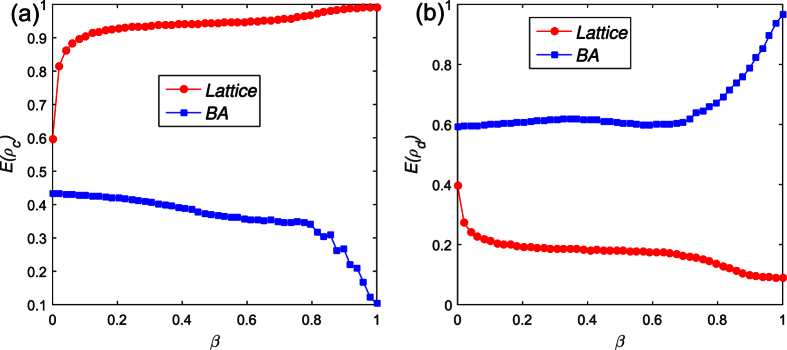
The average cooperator and death density change with the initial visibility level *β*. The average cooperator density increases and the average death density decreases with the increase of the initial level of visibility *β* in the lattice network. However, the contrary phenomenon is observed in the BA network. Parameters are set: *α* = 0.1, *r* = 1.1, *δ* = 0, *T* = 1.1, *κ* = 10. Each data point is obtained by averaging 100 independent runs from the previous *M* = 500 rounds.

With the increase of initial visibility level *β*, the average cooperator and death density show significantly different trend. As shown in Fig. 2(a), in the lattice network the average cooperator density increases with the initial visibility level *β*. However, the contrary phenomenon is observed in the BA network. On the other hand, we can also find from Fig. 2(b), in lattice network the average death density decreases with the initial level of visibility *β*. However, the contrary phenomenon is observed in the BA network. In the case of defection, more individuals are replaced for lack of resource. Hence, cooperation can effectively avoid death caused by the shortage of resources. In the lattice network, the largest degree of all players is 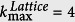. However, the largest degree in the BA network with the same of average degree 〈*k*〉 is 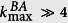 (for example, the largest degree is 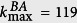 in the presented network, see the methods). If the node *i* with largest degree is a defector in the BA network, this player could obtain the expected number of resources far greater than it in the lattice network. Therefore, if the individuals with more neighbors in the BA network could know their neighbors payoff, those individuals are more willing to take betrayal to maximize their own payoff. With the increase of the initial visibility level *β*, the players with less neighbors are distributed few resource. Once when their resource *L*_*i*_ is less the critical value *δ*, those players will be replaced by others.

**Figure 3:**
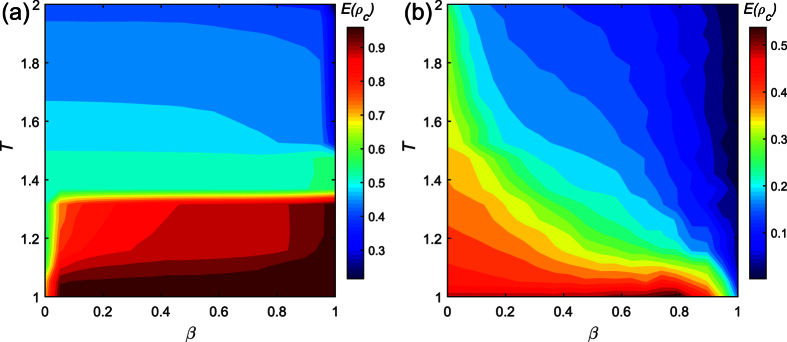
How the initial proportion of visibility *β* and the Successful-Defection-Payoff *T* affect the players’ strategies under the two kinds of networks structure. The expected payoff of players increase with the increase of the *T*, that contributes to the decline of average cooperator density *E*(*ρ*_*c*_), regardless of the network structure and individuals’ visibility. Parameters are set: *α* = 0.1, *r* = 1.1, *δ* = 0, *κ* = 10. Each data point is obtained by averaging 100 independent runs from the previous *M* = 500 rounds. (**a**) The average cooperator density changes with the *β* in the lattice network. (**b**) The average cooperator density changes with the *β* in the BA network.

The next, we detailed research how the initial proportion of visibility *β* and the Successful-Defection-Payoff *T* affect the individuals’ strategies. The expected payoff of players increase with the increase of the *T*, that contributes to the decline of average cooperator density *E*(*ρ*_*c*_), regardless of the network structure and individuals’ visibility (see the Fig. 3a,b). Moreover, the difference between maximum and minimum value *Max*(*E*(*ρ*_*c*_)) − *Min*(*E*(*ρ*_*c*_)) ≈ 0.8 in lattice network is bigger than in the BA network *Max*(*E*(*ρ*_*c*_)) − *Min*(*E*(*ρ*_*c*_)) ≈ 0.55. Therefore, the Successful-Defection-Payoff *T* has more influence for the players in lattice network than in BA network. A further comparative analysis could find the cooperation density in heterogeneous is less than in the homogenous network with the same *β* and *T*.

**Figure 4:**
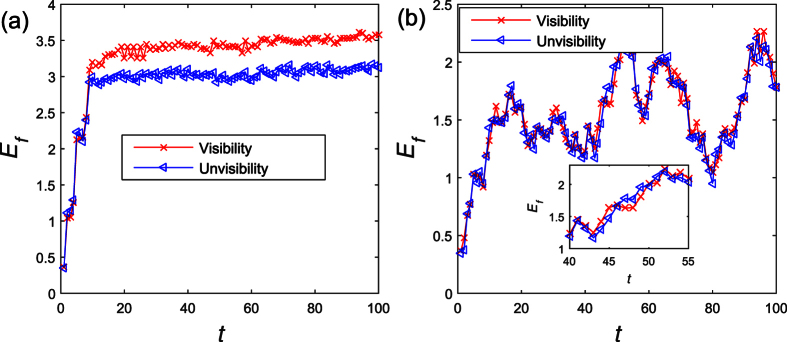
The average payoff of visibility and invisibility players change with the game round. The visibility individuals’ payoff *E*_*f*_ is larger than the invisibility individuals’ payoff *E*_*f*_ in the lattice network. However, the payoff of visibility and invisibility players are almost the same in the BA network. Parameters are set: *α* = 0.1, *β* = 0.5, *r* = 1.1, *δ* = 0, *T* = 1.1, *κ* = 10, *M* = 100. Each data point is obtained by averaging 100 independent runs. (**a**) The average payoff of visibility and invisibility players change with the game round in the lattice network. (**b**) The average payoff of visibility and invisibility players change with the game round in the BA network. The inset is a part of evolution of the visibility and invisibility individuals’ payoff in the BA network. We could find the visibility and invisibility individuals’ payoff change in the cross.

To better explore how the visibility affects the players’ payoff and strategies, we calculate the average payoff of the visibility and invisibility individuals in each game round. Figure 4(a,b) describe the individuals’ average payoff in the lattice network and BA network, respectively. From the Fig. 4(a), we might find the visibility individuals’ payoff *E*_*f*_ is larger than the invisibility individuals’ payoff *E*_*f*_ in the lattice network. Therefore, those invisibility individuals are easily to be replaced by the visibility individuals. However, the visibility players in homogenous incline to cooperate, that would raise the total number of cooperators. As a result, the visibility players would have a relative high payoff. On the other hand, as illustrated in Fig. 4(b), the average payoff of visibility and invisibility players are almost the same. In the BA network, the average visibility level *E*(*ρ*_*v*_) is almost linear with the initial visibility levels *β*. Therefore, the total payoff of the visibility players has a strong relation with the *β*. By comparison, the average payoff of player in the lattice network, regardless of their visibility, is larger than in the BA network. No matter whether the players are the visibility, adopted the defection strategy is the optimal selection in the BA network. However, the total payoff would be declined if most of the players are defectors. Therefore, the players could earn more in the lattice network.

**Figure 5:**
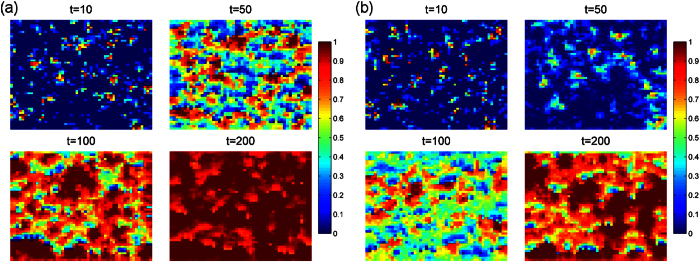
The evolution of the individuals’ visibility in the lattice network. The invisibility individuals are gradually replaced by the visibility individuals. Interestingly, the level of visibility declines with the increase of the Successful-Defection-Payoff *T*. Parameters are set: *α* = 0.1, *β* = 0.1, *r* = 1.1, *δ* = 0, *κ* = 10. Each data point is obtained by averaging 100 independent runs. (**a**) The Successful-Defection-Payoff is *T* = 1.1. (**b**) The Successful-Defection-Payoff is *T* = 1.3.

We plot the evolution of the individuals’ visibility in the lattice network (Fig. 5a,b). According to the timestamp of individuals’ visibility, it is easily to obvious that the invisibility individuals are gradually replaced by the visibility individuals. Interestingly, the level of visibility declines with the increase of Successful-Defection-Payoff *T*. The players incline to cooperate in the lattice network, therefore, the defectors could obtain more payoff if there exist a few defectors. Due to the limited resource, those individuals that connect to the defectors could only obtain relative small resource. As a result, they will be replaced by the offspring of the players with high payoff. However, if the Successful-Defection-Payoff *T* is relative larger, almost all the players choose to be the defectors. Therefore, the total resource are distributed to each player nearly equally, that leads to just a few players death. As a result, the growth of total number of visibility individuals will not be too big.

**Figure 6:**
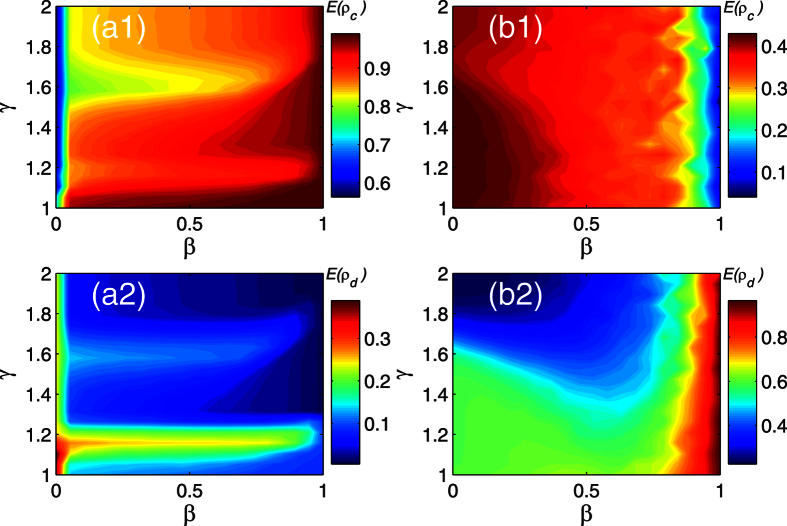
The average density of cooperator and death change with the initial visibility level *β* and environment parameter *γ*. Parameters are set: *α* = 0.1, *δ* = 0, *T* = 0.1, *κ* = 10, *M* = 500. Each data point is obtained by averaging 100 independent runs. (**a1**) The evolution of cooperator density in the lattice network. (**a2**) The evolution of death density in the lattice network. (**b1**) The evolution of cooperator density in the BA network. (**b2**) The evolution of death density in the BA network.

In general, a relative larger *r* means a rich and generous natural environment. Instead, a small *r* indicates the environment is very strictly. According to the Fig. 6(a1, we may find a rich environment could significantly hinder players cooperation in lattice network. As the surrounding environment becomes better, the betrayal strategy can bring more payoff, thus more individuals reluctant to cooperate. However, a rich environment is not the sufficient condition for decreasing the cooperators in the BA network. Moreover, a player might get less payoff, however, he could survive in a generous natural environment. Results presented in Fig. 6(a2 confirm that the effectiveness of a rich environment for preventing players to death.

**Figure 7:**
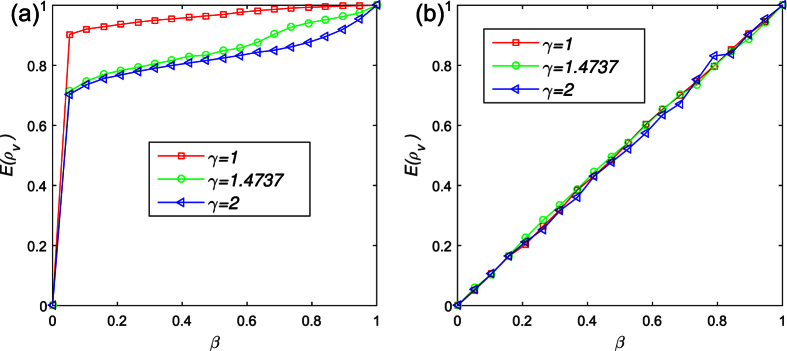
The average level of visibility changes with *β*. The initial visibility level *β* has greater influence on the lattice network than the BA network. While the individual connection in the form of lattice structure, the average level of visibility increases with the increase of the initial level of visibility *β*. However, the individual visibility level does not significantly improve if the connection in the form of BA network. Parameters are set: *α* = 0.1, *δ* = 0, *T* = 1.1, *κ* = 10, *M* = 500. Each data point is obtained by averaging 100 independent runs. (**a**) The average level of visibility changes with *β* in the lattice network. (**b**) The average level of visibility changes with *β* in the scale free network.

In the following, we study the visibility level evolution with the initial visibility level *β*. According to the results above, the initial visibility level *β* has great influence on the death, cooperation rate and individuals’ payoff. By comparison, the initial visibility level *β* has greater influence on the lattice network than BA network. While the individual connection in the form of lattice structure, the average level of visibility increases with the increase of the initial level of visibility *β* (see Fig. 7(a)). However, the individual visibility level does not significantly improve if the connection in the form of the BA network. Instead, *E*(*ρ*_*v*_) is almost linear with the initial visibility levels *β* (see Fig. 7(b)). It seemly that the changing of initial visibility has more influence of affect on the players in the homogeneous network. In addition, the average visibility level reduces with the increase of the environment parameter *γ*. The results further clarify the previous results: in the homogeneous network, the cooperation decreases with the increase of the initial proportion of cooperator *β*. However, in the heterogeneous network, with the increase of the initial proportion of cooperator *β* , the cooperation will not have a significant increase.

**Figure 8:**
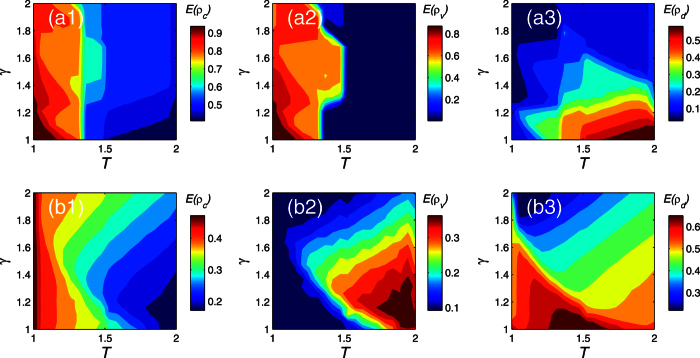
The density of cooperator, visibility and death evolution with the Successful-Defection-Payoff *T* and the environment parameter *γ*. Parameters are set: *α* = 0.1, *δ* = 0, *κ* = 10, *M* = 500. Each data point is obtained by averaging 100 independent runs. (**a1–a3**) The density of cooperator, visibility and death in the lattice network, respectively. (**b1–b3**) The density of cooperator, visibility and death in the BA network, respectively.

We may find from the Fig. 8(a1–a3, both in lattice network and in BA network, the density of cooperator decreases with the Successful-Defection-Payoff *T*. However, the changing of the density of visibility is quite different with the *T*. The increasing of *T* is beneficial to the level of individual visibility in heterogeneous networks. But it inhibits the individual visibility in the lattice networks. While the environment parameter *γ* is at a lower level, that is, the individuals are in a strict environment, the number of death in the BA network increase first and then down with the Successful-Defection-Payoff *T*. However, the number of death in the lattice network increases with the Successful-Defection-Payoff *T*.

## Discussion

“The most important unanswered question in evolutionary biology, and more generally in the social sciences, is how cooperative behavior evolved and can be maintained in human or other animal groups and societies” (Robert May in his Presidential Address to the Royal Society in 2005). Under the complexity of human mobility and interaction, the individual’s decision whether or not to cooperate can evolve in time mainly depending on their surroundings. In the real world, the environment condition impels the resource distributing to each individual is limited. In general, those individuals with a little resource have low fitness. According to the Darwin’s theory of evolution, the individuals whose fitness are extremely low would be replaced easily. Therefore, the principle of “survival of the fittest” is considered. On the other hand, some social experiments claimed that if people know all their neighbors’ information, people would not like to cooperate. Therefore, we consider how the initial visibility level and limited resource might affect the evolution of the players’ strategies. Based on the Monte Carlo methods, our results show that under the same level of initial visibility, players might have a higher expected payoff in homogeneous network than in heterogeneous network. Further, the average number of death decreases with the increases of the initial proportion of visibility in lattice network. However, we get the contrary results in the scale-free network. A high Successful-Defection-Payoff and a rich natural environment have relatively larger deleterious cooperation effects. The players are more likely to cooperate voluntarily in homogeneous network structure than in heterogeneous network structure. Our results verify the connection structure has great influence the individuals’ strategies.

This study offers a new perspective on the Prisoner’s dilemma game: how the initial level of visibility and limited resource affect the individuals’ strategies. Our analysis provides a novel thought for studying the co-evolution of games and strategies, and suggests that maintaining cooperation may be affected by many environment factors. In the real world, some restrictive assumptions may not be germane. However, it is worth emphasizing that our work shed light on the framework of evolutionary games on networks. Further experimental research is needed to clarify our results and we hope that our work will be motivational to that.

## Methods

### The network structure and simulation procedure

We perform our simulation in the lattice network and the scale-free network^30^. Each network contains 2500 nodes. The square lattice with periodic boundary condition is applied. The degree distribution of BA network is *P*(*k*) = 2 *m*^2^*k*^−*γ*^, where *m* denotes the minimum degree and *γ* is the power exponent. In order to facilitate to compare the results between the two networks, we set the average degree of the BA network is 〈*k*〉 = 2 *m* = 4. Monte Carlo methods are used to perform the prisoner’s dilemma game^31^,^32^. To alleviate the effect of randomly, each data point is obtained by averaging 100 independent runs.

### Calculation network diameter

Firstly, we use Floyd-Warshall algorithm to calculate the shortest path *d*_*ij*_ between any two nodes *i* and *j* in the given network topology. Then, the network diameter could be obtained: 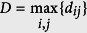.

### Calculation of the payoff

An individual with strategy *s*_*i*_ interacts with a *s*_*j*_ neighbor can acquire payoff *f*_*ij*_:


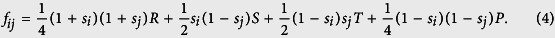


For simplicity but without loss of generality, the payoff matrix for the PDG is rescaled as *R* = 1, *S* = 0, *P* = 0. Thus, an individual with strategy *s*_*i*_ interacts with a *s*_*j*_ neighbor can acquire payoff *f*_*ij*_:


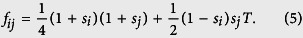


We can also calculate the total payoff *f*_*i*_ of player *i* as follows:


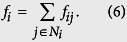


where *N*_*i*_ is neighbor set of node *i*.

### Calculation of statistical indices

Before the *M* game rounds, the average cooperator density, average invisibility density and death density are 
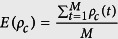
, 
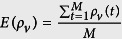
, 
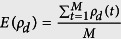
, respectively. *ρ*_*c*_(*t*), *ρ*_*v*_(*t*), *ρ*_*d*_(*t*) are the average cooperator density, average invisibility density and death density at the *t* game round. The average payoff of visibility and invisibility individuals are 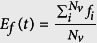 and 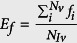, respectively. Where *N*_*v*_ and *N*_*Iv*_ are the number of visibility and invisibility individuals.

## Additional Information

**How to cite this article**: Han, D. *et al*. How the initial level of visibility and limited resource affect the evolution of cooperation. *Sci. Rep*. **6**, 27191; doi: 10.1038/srep27191 (2016).

## Figures and Tables

**Table 1:** The payoff matrix of the prisoner’s dilemma game.

Strategy	*s*_*j*_ = 1	*s*_*j*_ = −1
*s*_*i*_ = 1	(*R*, *R*)	(*S*, *T*)
*s*_*i*_ = −1	(*T*, *S*)	(*P*, *P*)
